# Characterization of enzymatic properties of two novel enzymes, 3,4-dihydroxyphenylacetate dioxygenase and 4-hydroxyphenylacetate 3-hydroxylase, from *Sulfobacillus acidophilus* TPY

**DOI:** 10.1186/s12866-019-1415-9

**Published:** 2019-02-13

**Authors:** Wenbin Guo, Wengen Zhou, Hongbo Zhou, Xinhua Chen

**Affiliations:** 1 Key Laboratory of Marine Biogenetic Resources, Third Institute of Oceanography, Ministry of Natural Resources, Xiamen, 361005 Fujian China; 2 School of Resource Processing and Bioengineering, Central South University, Changsha, 410083 Hunan China; 3 Institute of Oceanology, College of Animal Sciences, Fujian Agriculture and Forestry University, Fuzhou, 350002 Fujian China; 4 Fujian Key Laboratory of Marine Genetic Resources, Xiamen, 361005 China

**Keywords:** *Sulfobacillus acidophilus*, 4-hydroxyphenylacetate 3-hydroxygenase, 3,4-dihydroxyphenylacetate dioxygenase

## Abstract

**Background:**

As an environmental pollutant, 4-hydroxyphenylacetate (4-HPA) was a product of softwood lignin decomposition and was found in industrial effluents from olive oil production. *Sulfobacillus acidophilus* TPY was a moderately thermoacidophilic bacterium capable of degrading aromatic compounds including 4-HPA. The enzymes involved in the degradation of 4-HPA and the role of this strain in the bioremediation of marine pollutants need to be illustrated.

**Results:**

3,4-dihydroxyphenylacetate dioxygenase (DHPAO) encoded by *mhpB2* and two components of 4-hydroxydroxyphenylacetate (4-HPA) 3-hydroxylase encoded by *hpaB* and *hpaC* from *S. acidophilus* TPY, a moderately thermoacidophilic bacterium, involved in the degradation of 4-HPA possessed quite low amino acid sequence identity (22–53%) with other ever reported corresponding enzymes, which suggest their novelty. These two enzymes were expressed in *E. coli* and purified to homogeneity. DHPAO activity in *E. coli* was revealed by spraying with catechol or 3,4-dihydroxyphenylacetate (3,4-DHPA) on the colonies to make them turn brilliant yellow color. DHPAO possessed total activity of 7.81 U and 185.95 U/mg specific activity at the first minute when 3,4-DHPA was served as substrate. DHPAO was a thermophilic enzyme with optimum temperature of 50 °C and optimum substrate of 3,4-DHPA. The small component (HpaC) was a flavoprotein, and both HpaB and HpaC of 4-HPA 3-hydroxylase were NADH-dependent and essential in the conversion of 4-HPA to 3,4-DHPA. 4-HPA 3-hydroxylase possessed 3.59 U total activity and 27.37 U/mg specific activity at the first minute when enzymatic coupled assay with DHPAO was applied in the enzymatic determination.

**Conclusions:**

The ability of this extreme environmental marine strain to degrade catechol and substituted catechols suggest its applications in the bioremediation of catechol and substituted catechols polluted marine environments.

**Electronic supplementary material:**

The online version of this article (10.1186/s12866-019-1415-9) contains supplementary material, which is available to authorized users.

## Background

Catechol and substituted catechols were the key intermediate of the catabolic pathway of aromatic compounds [1]. Catabolism of phenolic compounds was often initiated by hydroxylases that incorporate hydroxyl groups into phenolic substrates, resulting in production of a few intermediates such as catechol and substituted catechols [2]. These intermediates then served as substrates for cleavage of the aromatic ring and could be further metabolized by two distinct sets of enzymes, which were responsible for the *ortho*- and *meta*- cleavage pathways [3]. The intermediate catechol could be catalyzed either by catechol 1,2-dioxygenase (C12O) to succinic acid and acetyl-CoA, which initiated the *ortho*-pathway [4, 5], or by catechol 2,3-dioxygenase (C23O) to pyruvate and acetaldehyde, which initiated the *meta*-pathway [6, 7]. Methyl-substituted aromatic substrates were generally degraded by C23O via *meta*- cleavage, whereas the ring cleavage of non-substituted aromatics and chloroaromatics trended to be cleaved through *ortho*- fission [8]. 3,4-dihydroxyphenylacetate (DHPA) was ususlly catalyzed by 3,4-Dihydroxyphenylacetate 2,3-dioxygenase (DHPAO), a mononuclear non-heme metal-containing enzyme, for the extradiol ring cleavage by incorporation of molecular oxygen to yield 5-carboxymethyl-2-hydroxymuconate semialdehyde (CHMS) [9].

C23O had strictly conserved residues responsible for binding of iron, and the Fe-binding sites were located in the C-terminal domain. The C23O from *Bacillus thermoleovorans* strain A2 was Fe^2+^-dependent and the Fe^2+^ was tightly bound, for the enzyme activity was not inhibited by EDTA but completely destroyed by H_2_O_2_ [10]. The metal cofactors of DHPAOs from different species show significant diversity and include Fe(II), Mn(II) and Mg(II) [9].

As an environmental pollutant, 4-hydroxyphenylacetate (4-HPA) was a product of softwood lignin decomposition [11], and was found in industrial effluents from olive oil production [12]. 4-HPA could be hydroxylated by bacteria via two routes with hydroxylation occurred at C-1 and C-3 respectively [13]. 4-HPA 3-hydroxylase was employed in the hydroxylation at C-3 of 4-HPA to produce 3,4-dihydroxyphenylacetate (3,4-DHPA) which then went through a *meta*-cleavage pathway with the final products of succinate and pyruvate [14]. *Pseudomonas putida*, *Acinetobacter* sp., *Pseudomonas ovalis* and *Escherichia coli* had all been shown to catabolize 4-HPA through the *meta*-cleavage route, with 3,4-DHPA being the hydroxylation product [15]. In the second pathway, hydroxylation occurred at C-1 of 4-HPA to generate homogentisate, which was initiated by 4-HPA 1-hydroxylase [16, 17]. The intermediate homogentisate was then converted into fumarate and acetoacetate [18].

4-HPA 3-hydroxylase was a two-protein component enzyme including a flavoprotein and a coupling protein [15]. The small component was a flavoprotein, which could bind to FAD and FMN, while the large component was the hydroxylase component, which was an absolute requirement for productive hydroxylation [19]. In *E. coli*, the large component encoded by *hpaB* showed only a very low hydroxylase activity in the absence of the small component encoded by *hpaC*, however, the hydroxylase activity was enhanced in the presence of the small protein [20]. As monitoring the oxidation of NADH was not sufficient to characterize 4-HPA 3-hydroxylase activity, 3,4-dihydroxyphenlacetate 2,3-dioxygenase (DHPAO) was used to measure the product formation activity of this enzyme [15].

*Sulfobacillus acidophilus* TPY was a moderately thermoacidophilic bacterium capable of degrading aromatic compounds. In this study, gene *mhpB2* (TPY_2461) encoding DHPAO and genes *hpaB* (TPY_2462) and *hpaC* (TPY_2460) encoding two components of 4-HPA 3-hydroxylase from *S. acidophilus* TPY were cloned and expressed in *E. coli*. The three proteins were purified and their enzymatic properties were characterized to reveal their function in the aromatic compound degradation of *S. acidophilus* TPY.

## Methods

### Bacterial strains and growth conditions

The bacterial strains used in this study were listed in Table 1. *Sulfobacillus acidophilus* TPY was isolated from a hydrothermal vent in the Pacific Ocean (12^°^42′29”N, 104^°^02′01”W; water depth: 3083 m) and cultivated on SA medium [21]. It was deposited in the China Center for Type Culture Collection (CCTCC) with the accession number CCTCC M 2010203. *Escherichia coli* strains were grown at 37 °C in Luria-Bertani (LB) medium or on LB agar plates supplemented with 100 μg/mL ampicillin or 50 μg/mL kanamycin, if necessary. *E. coli* strains were all commercialized strains purchased from commercial companies listed in Table 1.

**Table 1:** Bacterial strains, plasmids, and primers used in this study

Strains/plasmids/primers	Relevant characteristics	Reference/Source
Strains		
*Sulfobacillus acidophilus* TPY	Gram-positive, acidophilic, moderately thermophilic, isolated from a hydrothermal vent in the Pacific Ocean (12.2′29″N, 104.2′01″W; water depth 3083 m)	[28]
*E. coli* DH5α	F^−^, ∆(*lacZYA-argF*)*U169*, *recA1*, *endA1*, *hsdR17*(rk^−^, mk^+^), *supE44*, *thi-1*, *gyrA*, *relA1*	Invitrogen
*E. coli* DH5α (pET-32a(+) -hpaB)	*E. coli* DH5α harboring plasmid pET-32a(+)-hpaB, Ap^r^	This study
*E. coli* DH5α (pET-32a(+) -hpaC)	*E. coli* DH5α harboring plasmid pET-32a(+)-hpaC, Ap^r^	This study
*E. coli* DH5α (pET-28a(+) –mhpB2)	*E. coli* DH5α harboring plasmid pET-28a(+)–mhpB2, Ap^r^	This study
*E. coli* BL21 (DE3)	F^−^, *ompT*, *hsdS*(rBB^−^ mB^*−*^), *gal*, *dcm*(DE3)	Invitrogen
*E. coli* BL21 (DE3) (pET-28a(+)-mhpB2)	*E. coli* BL21 (DE3) harboring plasmid pET-28a(+)-mhpB2, Ap^r^	This study
*E. coli* BL21 (DE3) (pET-32a(+)-hpaB)	*E. coli* BL21 (DE3) harboring plasmid pET-32a(+)-hpaB, Ap^r^	This study
*E. coli* BL21 (DE3) (pET-32a(+)-hpaC)	*E. coli* BL21 (DE3) harboring plasmid pET-32a(+)-hpa**C**, Ap^r^	This study
Plasmids		
pET-28a(+)	Expression vector, Km^r^, His-tag	Takara
pET-32a(+)	Expression vector, Ap^r^, His-tag	Takara
pET-32a(+) -hpaB	Plasmid pET-32a(+) harboring *hpaB* gene, Ap^r^, His-tag	This study
pET-32a(+) -hpaC	Plasmid pET-32a(+) harboring *hpaC* gene, Ap^r^, His-tag	This study
pET-28a(+)-mhpB2	Plasmid pET-28a(+) harboring *mhpB2* gene, Km^r^, His-tag	This study
Primers	Sequence (5′-3′)	Restriction site
hpaB	F: 5′-AATGATATCATGGGGATTCGGACCGGCGAA-3′	*EcoR*V
	R: 5′-GGAAAGCTTTTACGACCGCGCCTCCTTTGT-3′	*Hind*III
hpaC	F: 5′-AATGATATCATGGGGATTCGGACCGGCGAA-3′	*EcoR*V
	R: 5′- GGAAAGCTTCTACCGTTCCAGTAACAAA-3′	*Hind*III
mhpB2	F: 5′-GGAATTCATGGGCGAAGCATTTAATAT-3′	*EcoR*I
	R: 5′-GGAAAGCTTTTAATCGTCAAGAAACGCCGG-3′	*Hind*III

*EcoR*V and *Hind*III recognition sites are underlined

### Amino acid sequence identity analysis of enzymes

Amino acid sequence identity analysis of DHPAO, HpaB and HpaC proteins of *S. acidophilus* TPY, encoded by *mhpB2* (TPY_2461), *hpaB* (TPY_2462) and *hpaC* (TPY_2460) respectively, were performed against the homologous enzymes in other bacteria using BioEdit software, resulting in the amino acid sequences identities between two enzymes.

### Gene cloning and construction of expression plasmid

The plasmids and primers used in this study were listed in Table 1. The *mhpB2*, *hpaB* and *hpaC* genes were amplified from *S. acidophilus* TPY genomic DNA, purified and digested with restriction enzymes, and ligated to pET-32a(+) or pET-28a(+) vector (Takara, Dalian, China) digested with the same restriction enzymes, respectively. The generated recombinant plasmids were named pET-32a(+)-hpaB, pET-32a(+)-hpaC and pET-28a(+)-mhpB2, and transformed into *E. coli* DH5α respectively. Positive colonies were selected on LB plates containing ampicillin or kanamycin, and confirmed by sequencing of the harbored plasmids. The recombinant plasmids were then transformed into *E. coli* BL21(DE3). The enzyme activity of positive transformants containing *mhpB2* gene was detected by spraying catechol (0.1 M) or 3,4-DHPA (0.025 M) solution on the cells grown on LB plates. Colonies of positive transformants overexpressing DHPAO would turn yellow on the plates, for the formation of yellow-colored product from colorless catechol [22] or 3,4-DHPA [3].

### Expression and purification of recombinant proteins

*Escherichia coli* BL21(DE3) cells harboring the plasmids pET-32a(+)-hpaB, pET-32a(+)-hpaC and pET-28a(+)-mhpB2 were cultivated at 37 °C in 100 mL of LB medium supplemented with 100 μg/mL ampicillin or 50 μg/mL kanamycin overnight respectively. Expression and purification of recombinant proteins were done according to our previous work [23]. The protein concentration was determined according to Bradford assay with bovine serum albumin as standard [24].

### Analysis of cofactor and electron donor preference of HpaC and HpaB using UV/visible spectroscopy

The UV/visible absorption spectrum of purified HpaC (500 μg/mL) was measured from 200 to 600 nm using a Shimadzu UV-1800 spectrophotometer to reveal its cofactor. The electron donor preference of HpaC and HpaB was assessed based on the oxidation of NADPH and NADH. Reactions were carried out at 25 °C in 200 μL mixtures containing 50 mM phosphate buffer (pH 8.0), 51 μg HpaC or 25 μg HpaB, and 98 μM NADH or 60 μM NADPH. The reactions were initiated by the addition of NAD(P)H. The oxidation of NAD(P)H by HpaC/HpaB was assessed spectrophotometrically from 190 to 800 nm over time to evaluate changes in the characteristic absorption peaks of the artificial electron donors.

### Determination of enzyme activity and substrate specificity of DHPAO

DHPAO catalyzes the conversion of catechol/ 3,4-DHPA to form 2-hydroxymuconic semialdehyde (HMS)/ 5-carboxymethyl-2-hydroxymuconic semialdehyde (CHMS) which are yellow compounds with λ_max_ at 375/ 380 nm. Reaction was carried out with 42.0 μg purified DHPAO in 50 mM Gly-NaOH buffer, pH 9.0 in a total volume of 2 mL at 25 °C. The reaction was initiated by the addition of 0.25 mM catechol or 3,4-DHPA. The degradation of catechol/ 3,4-DHPA and formation of HMS/ CHMS were assessed spectrophotometrically from 200 to 500 nm over time to evaluate changes in the characteristic absorption peaks of the chemical compounds. The control reaction with the absence of enzyme was also performed. One unit of enzyme was defined as formation of *meta*- cleavage compounds from catechol or its analogs that caused an optical density increase of 0.110 per min at 375 nm (3,4-DHPA at 380 nm) [25]. DHPAO activities towards various substituted catechols including pyrogallol, 3-methylcatechol, 3,4-dihydroxybenzoic acid, 3,4-dihydroxybenzaldehyde, tetrabromocatechol, phloroglucinol, 4-nitrocatechol, 4-methylcatechol were assayed using these substrates (0.25 mM) instead of catechol.

### Effect of pH, temperature, preservation condition and metal ions on DHPAO activity

The effect of pH on DHPAO activity was determined following the enzyme activity determination assay mentioned above at 25 °C in the pH range of 3.0–11.0 using the following buffers: 50 mM Tris-CH_3_COOH (pH 3.0–4.5), 50 mM phosphate buffer (pH 6.0–8.0), 50 mM Gly-NaOH (pH 9.0–11.0). On the determination of optimum catalytic temperature of DHPAO, reactions were carried out at 25–70 °C in 50 mM Gly-NaOH (pH 9.0). In order to investigate the effect of storage condition on DHPAO activity, the enzyme was stored at 25 °C, 4 °C and − 20 °C with or without 10% (*v*/v) acetone as long as 8 days. The enzyme activity of DHPAO was monitored every day. In order to reveal the effect of metal ions on the activity of DHPAO, pre-incubation of DHPAO in 50 mM Gly-NaOH buffer (pH 9.0) containing 10^− 4^ M metal ions including AgNO_3_ (10^− 5^ M), CoCl_2_·2H_2_O, BaCl_2_·2H_2_O, Pb(NO_3_)_2_, MnCl_2_·4H_2_O, MgCl_2_·6H_2_O, CuCl_2_·2H_2_O, NiSO_4_·6H_2_O, FeCl_3_·6H_2_O, CaCl_2_, ZnCl_2_, Al_2_(SO_4_)_3_·16H_2_O, and FeSO_4_·7H_2_O at 50 °C for 3 min was carried out which followed the method reported for C23O in *Variovorax* sp. 12S [26]. After that, the reactions were initiated by the addition of 0.25 mM catechol. H_2_O_2_ inhibition to DHPAO was also evaluated by the addition of 0.25% (*v*/v) H_2_O_2_.

### Determination of 4-HPA 3-hydroxylase activity using enzymatic coupled assay with DHPAO

Due to the deceptive substrate-dependent NADH oxidase activity of the flavoprotein component, 4-HPA 3-hydroxylase could not be assayed either by monitoring the substrate-dependent oxidation of NADH or by monitoring O_2_ consumption [15]. Hence, to demonstrate that 3,4-DHPA was the intermediate product of 4-hydroxyphenylacetic acid (4-HPA) degradation by 4-HPA 3-hydroxylase, DHPAO was utilized for the transformation 3,4-DHPA into 5-carboxymethyl-2-hydroxymuconic semialdehyde (CHMS). The characteristic yellow color product of CHMS presented a maximum absorption at 380 nm [27]. With a saturating amount of DHPAO, the rate of formation of CHMS represents the rate of formation of 3,4-DHPA due to 4-HPA 3-hydroxylase activity. The reaction mixture (2 mL) contained 50 mM phosphate buffer (pH 8.0), 280 μM NADH, purified HpaB, HpaC and MhpB2 to final concentrations of 40.1, 25.5 and 21.0 μg/mL, respectively. The reaction was initiated by the addition of 165 μM 4-HPA at 25 °C and sustained for 30 min. In order to further confirm the conversion of 4-HPA to 3,4-DHPA by 4-HPA 3-hydroxylase, reaction was carried out without the addition of DHPAO and the reaction mixture was detected by high resolution electrospray ionization mass spectrometry (HR-ESI-MS) analysis which was performed on a quadrupole-time of flight mass spectrometer (Waters Xevo G2-XS QTof, USA).

## Results

### Analysis of amino acid sequence homology of DHPAO and two 4-HPA 3-hydroxylase components HpaB and HpaC from S. Acidophilus TPY

Amino acid (a.a.) sequence alignments of DHPAO and two 4-HPA 3-hydroxylase components HpaB and HpaC from *S. acidophilus* TPY were performed against homologous enzymes in other bacteria respectively. DHPAO of *S. acidophilus* TPY displayed the highest a.a. identity of 26% with C23O of *Geobacillus thermoleovorans* A2, while the two 4-HPA 3-hydroxylase components HpaB and HpaC of *S. acidophilus* TPY showed significant a.a. identities of 53% to HpaB of *Thermus thermophiles* and 42% to phenol hydroxylase small subunit of *Geobacillus stearothermophilus* (Table 2). Although the a.a. identity of DHPAO from *S. acidophilus* TPY was significantly low with other corresponding enzymes, the putative metal ligand binding residues His-146, His-209 and Glu-262, the catalytic residues His-195, His-243 and Tyr-252 and three other residues Gly-32, Leu-165, Pro-256 were strictly conserved with other extradiol dioxygenases (Additional file 1: Figure S1). These results indicated that DHPAO and 4-HPA 3-hydroxylase from *S. acidophilus* TPY were potential novel extradiol dioxygenase and two-component aromatic hydroxylases in a.a. sequence, respectively.

**Table 2:** Homology comparision of DHPAO, 4-HPA 3-hydroxylase components HpaB and HpaC from *S. acidophilus* TPY with homologous enzymes in other bacteria

Gene name (Locus Tag)	Homologous gene	Bacterium	Function	a.a. Identity	Reference^a^
*mhpB2* (TPY_2461)	*afpB*	*Alcaligenes faecalis IS-46*	catechol 2,3-dioxygenase	27%	ABQ14527
	*aphB*	*Comamonas testosteroni* TA441		26%	BAA34176
	*pheB*	*Geobacillus thermoleovorans* A2		30%	AF031325
	*dmpB*	*Pseudomonas* sp. CF600		29%	BAP28473
	*phnE2*	*Burkholderia* sp. RP007		27%	AAF02430
	Unknow	*Achromobacter xylosoxidans strain* LHB21		29%	GU199432
	xylE	*Stenotrophomonas maltophilia strain* KB2		29%	EF694961
	*Unknow*	*Limnobacter sp.* 2D3		30%	AFS60573
	*cdoE*	*Comamonas sp.* JS765		26%	U93090
	*pheB*	*Geobacillus stearothermophilus*		30%	P31003
	Unknow	*Geobacillus sp.* JF8		30%	WP_020961579
	Unknow	*Bacillus azotoformans*		27%	WP_004432276
*hpaB* (TPY_2462)	*hpaB*	*Escherichia coli*	4-HPA 3-hydroxylase large component	30%	Z29081
	*C2-hpah*	*Acinetobacter baumannii*	p-HPA hydroxylase C2: oxygenase component	–	AY566612
	hpaA	*Klebsiella pneumoniae*	4-HPA-3-hydroxylase	30%	L41068
	hpaB	*Thermus thermophilus*	4-HPA-3-hydroxylase	53%	BAD70783
	*pheA1*	*Rhodococcus erythropolis*	phenol hydroxylase large subunit	31%	ABS30825
	pheA1	*Geobacillus stearothermophilus*	phenol-hydroxylase large subunit	–	DQ146476
		*ssstearothermophilus*			
	pheA1	*Bacillus thermoglucosidasius*	Phenol 2-hydroxylase component A	32%	AF140605
	*hpaB*	*Shigella flexneri*	4-HPA 3-monooxygenase oxygenase component	30%	EIQ15487
*hpaC* (TPY_2460)	*hpaC*	*Escherichia coli*	4-HPA 3-hydroxylase small subunit component	27%	Z29081
	*C1-hpah*	*Acinetobacter baumannii*	p-HPA hydroxylase C1: reductase component	38%	AY566613
	unknow	*Klebsiella pneumoniae*	coupling protein	22%	L41068
	unknow	*Thermus thermophilus*	Conserved hypothetical protein	35%	BAD70784
	*pheA2*	*Rhodococcus erythropolis*	phenol hydroxylase small subunit	38%	ABS30826
	*pheA2*	*Geobacillus stearothermophilus*	phenol hydroxylase small subunit	42%	DQ146476
	*pheA2*	*Bacillus thermoglucosidasius*	Phenol 2-hydroxylase component B	38%	AF140605
	*hpaC*	*Shigella flexneri*	4-HPA 3-monooxygenase reductase component	23%	EIQ15486

^a^NCBI accession numbe

### Expression and purification of recombinant DHPAO, HpaC and HpaB

The recombinant DHPAO encoded by *mhpB2* and recombinant 4-HPA 3-hydroxylase encoded by *hpaC* and *hpaB* were primarily present as soluble proteins in the supernatants. The His-tagged recombinant proteins were then purified by Ni-affinity chromatography. The eluate containing His-tagged HpaC was brownish-yellow in color, indicating that the prosthetic group was still bound to the protein. The UV-visible spectrum of purified HpaC showed absorbance peaks at around 280, 412 and 452 nm (arrows), with shoulders at around 390 and 480 nm (Additional file 2: Figure S2). These absorbance peaks were attributed to the flavin (absorbance maxima at about 280, 375 and 450 nm) bound to HpaC. SDS-PAGE analysis revealed that the purified proteins were all exhibiting a single band with molecular mass of 35, 39 and 55 kDa for HpaC, DHPAO and HpaB, respectively (Additional file 3: Figure S3).

### Identification of DHPAO activity in *E. coli*

**Fig. 1:**
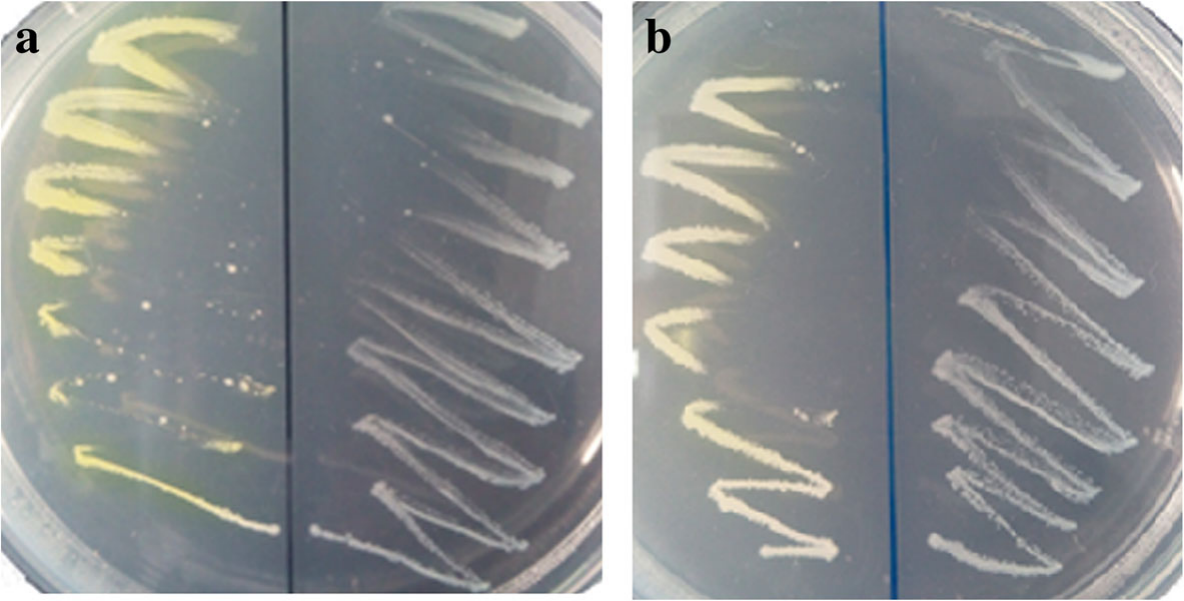
Identification of DHPAO activity in *E. coli* by spraying the plates with 0.1 M catechol (**a**) or 0.025 M 3,4-DHPA (**b**)

In this study, *E. coli* BL21(DE3) was transformed with recombinant plasmids pET-28a(+)-mhpB2 and pET-28a(+) as a control. Then the cultures were grown on LB plate with 50 μg/mL kanamycin at 37 °C overnight. When the plate was sprayed with 0.1 M catechol, the culture with recombinant plasmid pET-28a(+)-mhpB2 showed brilliant yellow color as the accumulation of 2-HMS (Fig. 1a, left), while the culture harboring plasmid pET-28a(+) did not change in color (Fig. 1a, right). Similarly, when sprayed with 0.025 M 3,4-DHPA, the culture harboring plasmid pET-28a(+)-mhpB2 turned yellow as the accumulation of CHMS (Fig. 1b, left), while the culture with plasmid pET-28a(+) didn’t (Fig. 1b, right). These results indicated that DHPAO encoded by *mhpB2* from *S. acidophilus* TPY was successfully expressed in *E. coli* BL21(DE3) with activity.

### Enzymatic activity of DHPAO

**Fig. 2:**
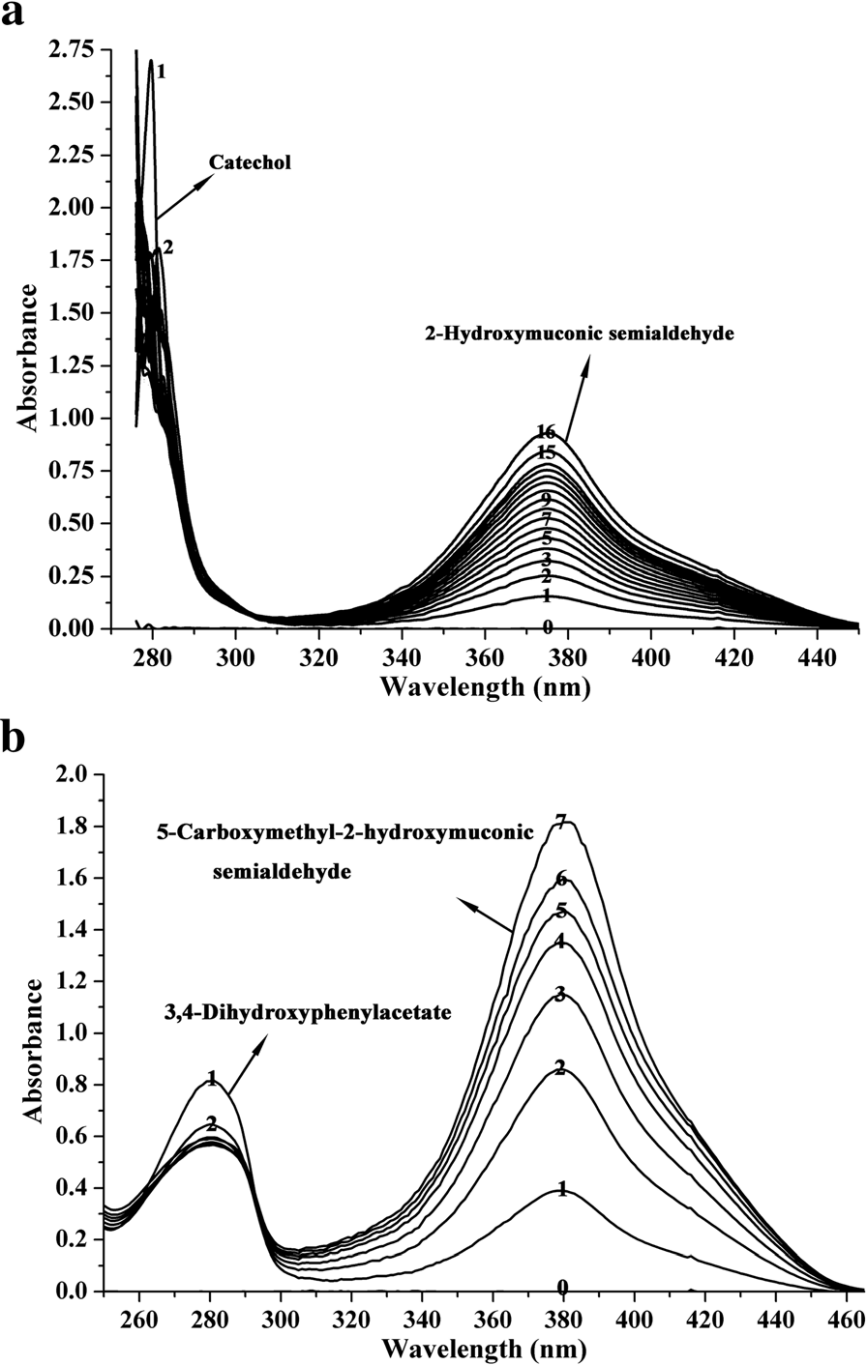
Enzymatic conversion of catechol to 2-HMS (**a**) and 3,4-DHPA to CHMS (**b**) by DHPAO. a, For catechol, spectra were recorded at 0 s (0, before adding the substrate), 30 s (1), 1 min (2), 1 min 30 s (3), 2 min (4), 2 min 30 s (5), 3 min (6), 3 min 45 s (7), 4 min 30 s (8), 5 min 30 s (9), 6 min 30 s (10), 8 min (11), 9 min 30 s (12), 11 min 30 s (13), 14 min (14), 19 min (15), 29 min (16). b, For 3,4-DHPA, spectra were recorded at 0 s (0, before adding the substrate), 30 s (1), 1 min (2), 1 min 30 s (3), 2 min (4), 2 min 45 s (5), 3 min 45 s (6), 8 min 45 s (7)

In addition to the catechol and 3,4-DHPA spraying assay in the investigation of recombinant DHPAO activity in *E. coli*, the enzymatic activity of the purified DHPAO was also determined by the absorption changes during the enzymatic conversion of catechol and 3,4-DHPA. The enzymatic reaction was initiated with the addition of substrates. When catechol was used as substrate, an increase at the maximal absorption of 375 nm was observed, which was due to the product 2-HMS (Fig. 2a). An optical density increase of 0.253 at the first minute indicated DHPAO possessed 2.30 U total activity and 54.76 U/mg specific activity at the first minute when catechol was served as substrate. At the same time, the decrease of catechol absorption at 280 nm was also detected. Besides, when 3,4-DHPA was served as substrate, an increase at the maximal absorption of 380 nm was also observed due to the production of CHMS. At the same time of CHMS formation, which caused an optical density increase of 0.859 at the first minute, the decrease of 3,4-DHPA was also appeared (Fig. 2b). This indicated DHPAO possessed total activity of 7.81 U and 185.95 U/mg specific activity at the first minute when 3,4-DHPA was served as substrate.

### Substrate range of DHPAO

In addition to catechol and 3,4-DHPA, other substituted catechols were served as the substrates to investigated DHPAO activities towards them. DHPAO exhibited a broad substrate specificity that pyrogallol, 3-methylcatechol, 4-methylcatechol, 3,4-dihydroxybenzoic acid, 3,4-dihydroxybenzaldehyde, tetrabromocatechol also could be utilized as substrate by DHPAO (Table 3). The relative activity of DHPAO towards catechol was set as 100%. DHPAO had higher ring-fission activity towards 4-methylcatechol (109.17% relative activity) than 3-methylcatechol (76.87% relative activity). Furthermore, DHPAO exhibited the highest enzyme activity towards 3,4-DHPA (212.36% relative activity), which was almost 2.1- fold of the enzyme activity towards catechol. No degradation of phloroglucinol and 4-nitrocatechol was observed. Meanwhile, 3,4-dihydroxybenzaldehyde and tetrabromocatechol degradation almost could not be detected. Pyrogallol and 3,4-dihydroxybenzoic acid had 28.42 and 33.09% relative activity respectively indicating that they could not be easily utilized by DHPAO.

**Table 3:** Substrate specificity of DHPAO

Substrates	Relative activity (%)
Catechol	100
Pyrogallol	28.42 ± 1.69
3-Methylcatechol	76.87 ± 2.55
4-Methylcatechol	109.17 ± 0.32
3,4-Dihydroxybenzoic acid	33.09 ± 1.37
3,4-Dihydroxyphenylacetic acid	212.36 ± 1.55
3,4-Dihydroxybenzaldehyde	<1
Tetrabromocatechol	<1
Phloroglucinol	–
4-Nitrocatechol	–

### Effects of pH, temperature and preservation conditions on DHPAO activity towards catechol

**Fig. 3:**
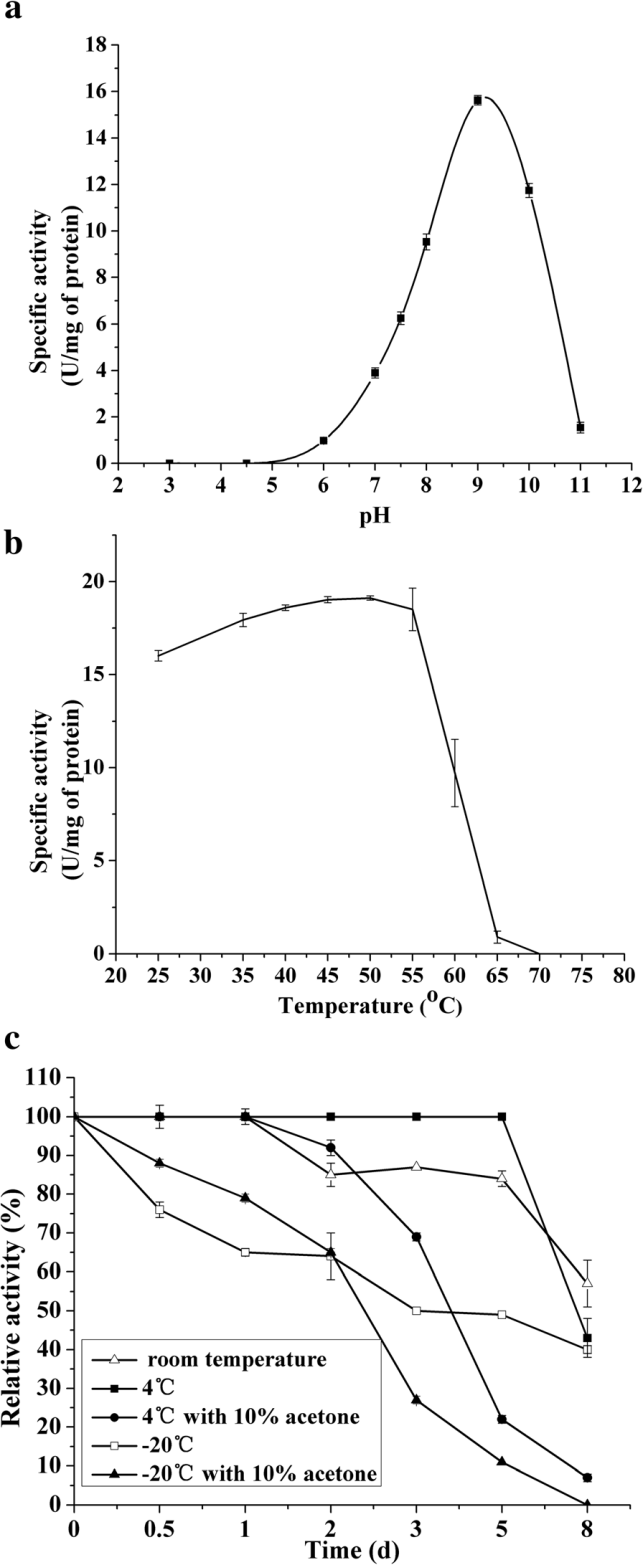
Effects of pH (**a**), temperature (**b**) and preservation method (**c**) on DHPAO activity

To evaluate the effect of pH on the DHPAO reaction activity towards catechol, the enzyme activity was measured at various pH conditions (3.0–11.0). The pH-specific activity profile showed a bell-shaped curve which indicated an optimum pH of 9.0 for the DHPAO reaction (Fig. 3a). In pH 9.0, the maximal specific activity of the enzyme was 15.623 ± 0.196 U/mg. However, when the pH was 6.0 and 11.0, the specific activities of the enzyme were reduced to 0.977 ± 0.042 U/mg and 1.540 ± 0.236 U/mg respectively (Fig. 3a). The enzyme lost its catalytic activity completely in pH 4.0 and 12.0 (Fig. 3a). The specific activities of DHPAO at different temperatures were evaluated in the optimum pH 9.0. The results indicated that the catalytic activity of DHPAO increased upon increasing the temperature up to 50 °C and then decreased at higher temperatures (50–70 °C) (Fig. 3b). The maximal specific activity of the enzyme was 19.113 ± 0.112 U/mg at 50 °C (Fig. 3b). The enzyme showed a broad temperature range of high enzyme activity between 35 °C and 55 °C (Fig. 3b). The data suggest that DHPAO was a thermostable enzyme that could be useful for future applications in biotechnology.

Many storage conditions were used to investigate the preservation of DHPAO activity. When DHPAO was kept at 4 °C, the enzyme activity maintained at 100% relative activity in the first 5 days and decreased to 43% relative activity in the eighth day (Fig. 3c). When DHPAO was stored at room temperature (25 °C) and − 20 °C, the enzyme activity decreased gradually from 1 to 8 day with 60 and 43% relative activity remained at the eighth day, respectively (Fig. 3c). When DHPAO was preserved at 4 °C and − 20 °C with the addition of 10% (*v*/v) acetone, the enzyme activity lost more quickly than that without acetone at the same temperature and almost disappeared at the eighth day (Fig. 3c). In a word, storage of DHPAO at 4 °C without acetone was the optimal preservation condition.

### Effects of metal ions and H_2_O_2_ on DHPAO activity

The effects of metal ions on DHPAO activity were shown in Additional file 4: Table S1. This enzyme was slightly inhibited by Ag^2+^, Co^2+^ and Fe^2+^ with 90.58–93.17% relative activity remained, seriously inhibited by Mn^2+^, Al^3+^, and Cu^2+^ with only 19.05, 67.13 and 6.37% relative activity remained, and promoted by Ba^2+^, Pb^2+^, Mg^2+^, Ni^2+^, Fe^3+^, Ca^2+^, Zn^2+^ with 103.57–131.16% relative activity obtained. Furthermore, DHPAO was completely inhibited by the addition of 0.25% (v/v) H_2_O_2_.

### NADH is the preferred electron donor of HpaB and HpaC

**Fig. 4:**
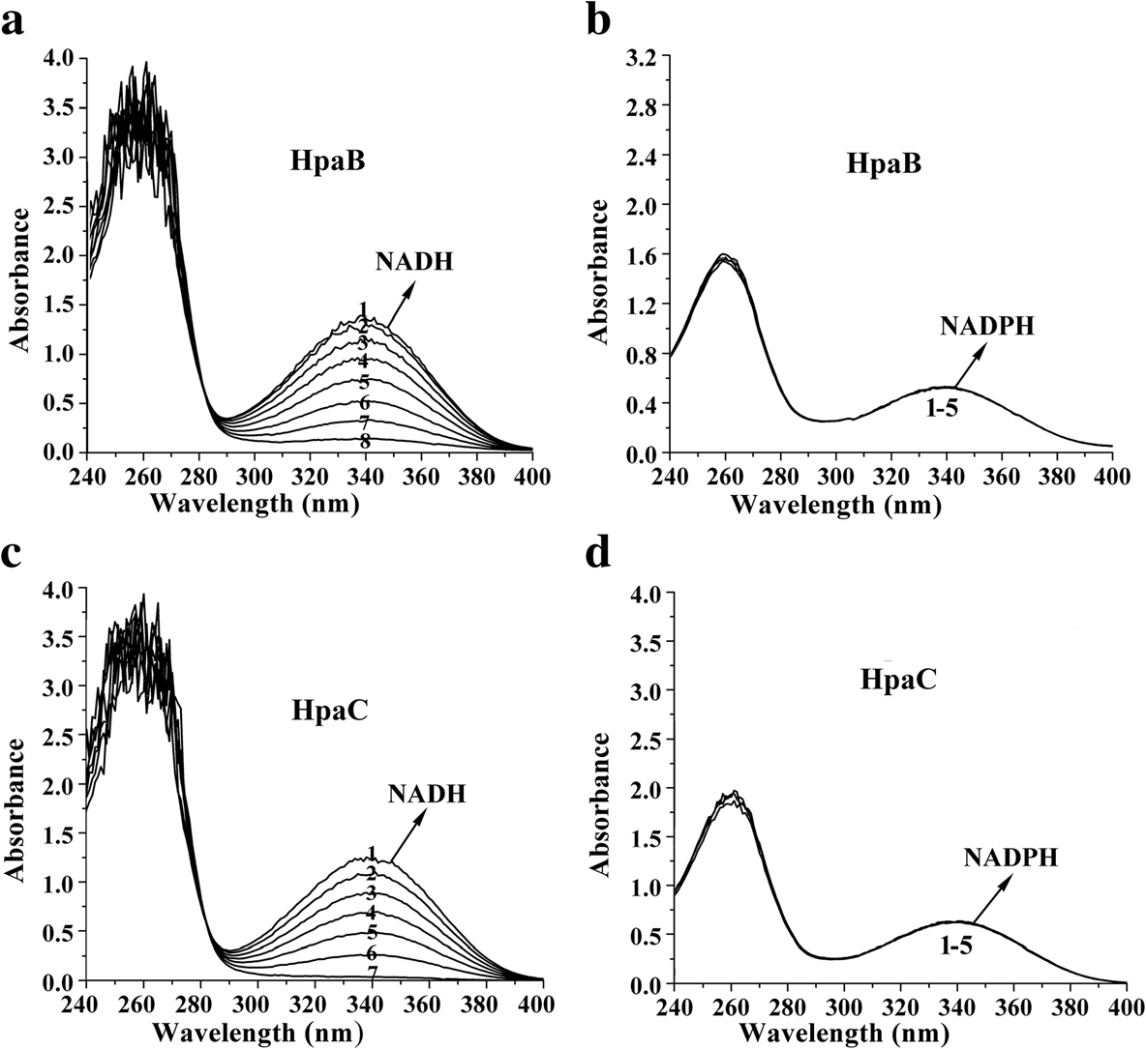
Investigation of NADH (a, c) and NADPH (b, d) utilization preference by HpaB (**a**, **b**) and HpaC (**c**, **d**). Spectra were recorded at 40 s (1), 2 min (2), 2 min 30 s (3), 3 min (4), 3 min 30 s (5), 4 min (6), 5 min (7) and 7 min 30 s (8)

In this study, the preference of using NADH or NADPH as the electron donor by HpaB and HpaC was evaluated. HpaB and HpaC exhibited no electron transfer when NADPH was used as the electron donor, but high electron transfer activity was observed with NADH as the electron donor (Fig. 4). Changes in the absorption peaks of UV-visible spectra were monitored during the oxidation of NADH by HpaB and HpaC. As shown in Fig. 4a, c, the characteristic absorption of the reduced form, NADH, decreased at 340 nm over the time course of the reactions.

### 4-HPA 3-hydroxylase activity

**Fig. 5:**
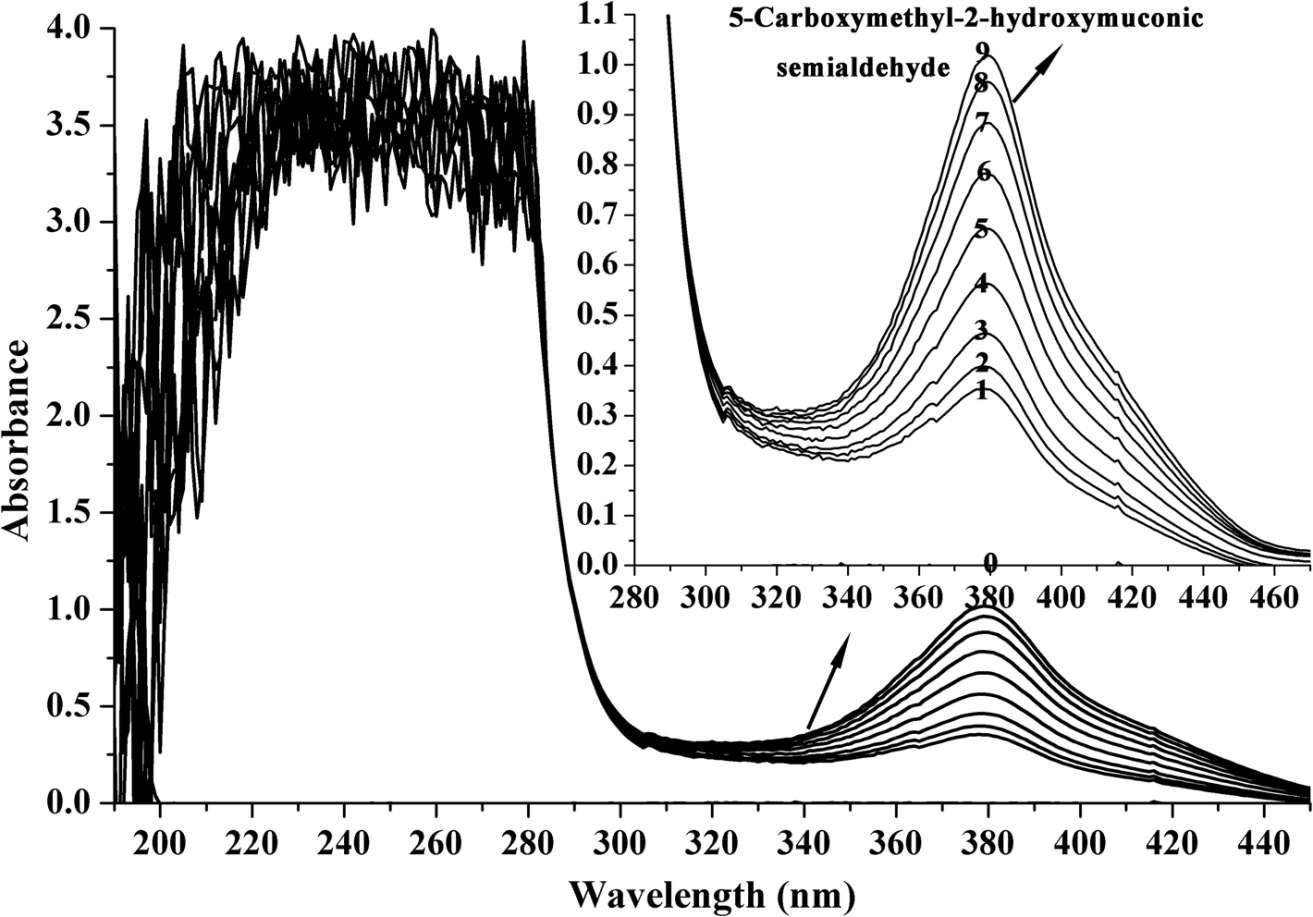
Detection of enzymatic coupled conversion of 4-HPA to CHMS by 4-HPA 3-hydroxylase and DHPAO. Spectra were recorded at 0.5 min (1), 1 min (2), 5 min (3), 10 min (4), 15 min (5), 20 min (6), 25 min (7), 30 min (8) and 40 min (9)

In the determination of 4-HPA 3-hydroxylase activity using enzymatic coupled assay with DHPAO, no activity was detected in the absent of HpaB or HpaC. This indicated that the two components HpaB and HpaC were all required for 4-HPA 3-hydroxylase activity. NADH was also required for 4-HPA 3-hydroxylase activity. The coupled enzymatic conversion of 4-HPA to final product of CHMS by 4-HPA 3-hydroxylase and DHPAO was shown in Fig. 5. Increase in the absorbance at 380 nm was observed indicating the formation of final product CHMS. An optical density increase of 0.395 at the first minute indicated 4-HPA 3-hydroxylase possessed 3.59 U total activity and 27.37 U/mg specific activity at the first minute. The HR-ESI-MS data at 167.0333 confirmed the product 3,4-DHPA (calcd. For *m/z* 167.0344 [M-H]^−^) of 4-HPA 3-hydroxylase (Additional file 5: Figure S4).

## Discussion

*S. acidophilus* TPY was reported to be capable of degrading phenol at 45 °C and pH 1.8 via the *meta*-pathway [28]. Except C23O encoded by *mhpB* gene [28], DHPAO encoded by *mhpB2* gene in this study might be highly possible involved in the degradation of catechol which was an important degradation intermediate of phenol. Gene *mhpB* was found in the phenol degradation cluster (TPY_0628–0634), while *mhpB2* (TPY_2461) was flanked by *hpaB* (TPY_2462) and *hpaC* (TPY_2460). Therefore, *mhpB2* encoding DHPAO, and *hpaB* and *hpaC* encoding two components of 4-HPA 3-hydroxylase from *S. acidophilus* TPY were cloned and expressed in *E. coli*, and the corresponding enzymes were purified and characterized. DHPAO, HpaB and HpaC were characterized as novel enzymes with significantly low amino acid sequence identity (22–53%) with other ever reported corresponding enzymes and distinct enzymatic properties. To the best of our knowledge, this is the first report of expression and characterization of novel DHPAO and 4-HPA 3-hydroxylase from *S. acidophilus*. These results will facilitate further understanding of the enzymes involved in the aromatic compound degradation in moderately thermoacidophilic *S. acidophilus*.

IPTG was not necessary in the expression of the three enzymes in *E. coli*. Similarly, IPTG induction caused no significant increase in protein expression had been reported [29]. The three enzymes showed higher molecular mass in the SDS-PAGE gel than the theoretical values which may due to the His-tag in the recombinant proteins (Additional file 3: Figure S3). The recombinant DHPAO activity in *E. coli* was first confirmed by a chromogenic identification (Fig. 1) using catechol spraying method, which was generally utilized for identifying dioxygenase activity [22, 29]. HpaB and HpaC coupled with DHPAO were used in the determination of 4-HPA 3-hydroxylase activity in which excessive amount of DHPAO was used to make sure that all the intermediate 3,4-DHPA produced could be transformed to CHMS. NADH was essential for the recombinant 4-HPA 3-hydroxylase activity. Some domains of 4-HPA 3-hydroxylase of *S. acidophilus* TPY might be essential for its enzyme activity. It had been reported that the Phe-216 in C-terminal Domain of 4-Hydroxyphenylacetate 3-Hydroxylase from *Acinetobacter baumannii* was important in the 4-HPA-stimulated NADH oxidase activity [30]. Differently, extracts from *Klebsiella pneumoniae* cells grown on 3-HPA showed 3-HPA hydroxylase activity using either NADH or NADPH as cofactor [14].

The optimum temperature of DHPAO from *S. acidophilus* TPY was 50 °C which was higher than C23O (40 °C) from *Pseudomonas* sp. strain ZJF08 [31]. This was not unexpected as *S. acidophilus* TPY was a moderately thermoacidophilic Gram-positive bacterium isolated from a hydrothermal vent in the Pacific Ocean with optimum growth temperature of 45 °C [28]. The optimum pH of DHPAO from *S. acidophilus* TPY was 9.0 which was higher than the optimum pH (7.5) of DHPAO from *Pseudomonas aeruginosa*. Although the optimum pH of chlorocatechol 2,3-dioxygenase from *Pseudomonas. putida* GJ31 was 9.6, the enzyme was unstable at this pH [32]. In this study, DHPAO remained almost total of its original activity within 5 days when it was stored in the presence of air at 4 °C. However, the C23O from *Pseudomonas* sp. OC1 was easily inactivated in the presence of air, owing to its sensitivity to oxygen [25]. Acetone was ever used as a protective agent to safeguard the enzyme from inactivation [1, 33]. However, addition of acetone in this study couldn’t protect DHPAO activity, but decrease the enzyme activity faster (Fig. 3). It was speculated that acetone served as a competitive inhibitor against catechol [34].

It was reported that intradiol-cleaving catechol dioxygenase typically contain ferric ion in the catalytic active metal center, while extradiol enzymes generally require the ferrous ion [35]. In this study, ferric ion seemed more important than ferrous ion for this extradiol enzyme DHPAO which was slightly inhibited by Fe^2+^ with 92.34% relative activity remained and promoted by Fe^3+^ with 114.03% relative activity obtained. It was reported that C23O1 exhibited the highest enzyme activity (265.65%) with Fe^2+^ added, while C23O2 showed the largest activity (377.37%) in the presence of Fe^3+^, although the two C23Os were from the same strain, a halophilic bacterial consortium (HF-1) [36]. Except ferrous ion and ferric ion, Mn^2+^ was reported to be necessary for the extradiol dioxygenase from *Bacillus brevis* [37]. However, the enzymatic activity of DHPAO from *S. acidophilus* TPY was seriously inhibited by Mn^2+^ with only 19.05% relative activity remained. In this study, aromatic ring-fission activities of the DHPAO towards catechol and catechol analogue were 3,4-Dihydroxyphenylacetic acid > 4-methylcatechol > catechol > 3-methylcatechol > 3,4-Dihydroxybenzoic acid > Pyrogallol. Diverse substrate preferences were observed in C23O from other strains. C23O from *Stenotrophomonas maltophilia* KB2 also reported to have a wide spectrum of aromatic substrates [38]. The C23O from archaeon *Sulfolobus solfataricus* strain 98/2 showed the highest ring-fission activity to catechol [39]. In halophilic bacterial consortium (HF-1), C23O1 had the highest ring-fission activity to catechol, while C23O2 possessed the largest ring-fission activity to 4-chlorocatechol [36].

## Conclusions

In this study, 3,4-dihydroxyphenylacetate (DHPA) dioxygenase (DHPAO) encoded by *mhpB2* and two components of 4-hydroxydroxyphenylacetate (4-HPA) 3-hydroxylase encoded by *hpaB* and *hpaC* from *S. acidophilus* TPY involved in the degradation of 4-HPA were expressed in *E. coli* and characterized as novel enzymes with significantly low amino acid sequence identity (22–53%) and distinct enzymatic properties. It is important to investigate the enzymes involved in aromatic compounds degradation pathway in this autotrophic and facultative heterotrophic microorganism and consider it in the application of bioremediation of catechol and substituted catechols polluted marine environments.

## Additional files

Additional file 1: **Figure S1**. Amino acid sequences alignment of MhpB2 with other extradiol dioxygenases. (DOCX 280 kb)

Additional file 2: **Figure S2**. UV-visible absorption spectrum of HpaC. (DOCX 188 kb)

Additional file 3: **Figure S3**. SDS-PAGE analysis of purified proteins. (DOCX 891 kb)

Additional file 4: **Table S1**. The effect of metal ions on DHPAO activity. (DOCX 17 kb)

Additional file 5: **Figure S4**. Mass spectrum of 3,4-DHPA transformed from 4-HPA by 4-HPA 3-hydroxylase. (DOCX 77 kb)

## Abbreviations

3,4-DHPA: 3,4-dihydroxyphenylacetate
4-HPA: 4-hydroxydroxyphenylacetate
C12O: catechol 1,2-dioxygenase
C23O: catechol 2,3-dioxygenase
CHMS: 5-carboxymethyl-2-hydroxymuconic semialdehyde
DHPAO: 3,4-dihydroxyphenylacetate dioxygenase
HMS: 2-hydroxymuconic semialdehyde
HR-ESI-MS: High resolution electrospray ionization mass spectrometry
