# Supplementary material for: Characterization of enzymatic properties of two novel enzymes, 3,4-dihydroxyphenylacetate dioxygenase and 4-hydroxyphenylacetate 3-hydroxylase, from Sulfobacillus acidophilus TPY
Source: BMC Microbiol. 2019 Feb 13;19:40. doi: 10.1186/s12866-019-1415-9 (PMC6375179; doi:10.1186/s12866-019-1415-9)
Supplement: Supplementary file 1 — Figure S1. Amino acid sequences alignment of MhpB2 with other extradiol dioxygenases. (DOCX 280 kb) [file 12866_2019_1415_MOESM1_ESM.docx]

**

**

**Fig. S1.** Amino acid sequences alignment of MhpB2 with other extradiol dioxygenases.

Residues conserved in all proteins are indicated by asterisks, the strictly conserved residues are given in bold number. Numbering refers to the sequence of MhpB2. The sequences are as follows: MhpB2, 3,4-dihydroxyphenylacetate dioxygenase from *Sulfobacillus acidophilus* TPY (GenBank accession numbers: AEJ40621); HpaD, 3,4-dihydroxyphenylacetate 2,3-dioxygenase from *Geobacillus stearothermophilus* (WP_043905972); MndD, 3,4-dihydroxyphenylacetate 2,3-dioxygenase from *Arthrobacter globiformis* CM-2 (U19817.1); PheB, catechol-2,3-dioxygenase from *Geobacillus stearothermophilus* (P31003); Cdo, catechol 2,3-dioxygenase from *Rhodococcus rhodochrous* CTM (X69504.1); DmpB, catechol 2,3-dioxygenase from *Pseudomonas* sp. CF600 (BAP28473.1); KB35B, catechol 2,3-dioxygenase from *Pseudomonas* sp. KB35B (ABB72208.1); TdnC, 3-methylcatechol 2,3-dioxygenase from *P. putida* UCC2 (X59790); AphB, catechol 2,3-dioxygenase from *Comamonas testosterone* TA441 (BAA34176); KF715, 2,3-dihydroxybiphenyl dioxygenase from *P. putida* KF715 (M33813.1); BphC, 2,3-dihydroxybiphenyl dioxygenase from *Pseudomonas* sp. strain LB400 (X66122); CumC, catechol 2,3-dioxygenase from *Pseudomonas fluorescens* IPO1 (D37828.1); TodE, 3-methylcatechol 2,3-dioxygenase from *Pseudomonas* *putida* (AAA26010.1).
