# Supplementary figures and images for: Characterization of enzymatic properties of two novel enzymes, 3,4-dihydroxyphenylacetate dioxygenase and 4-hydroxyphenylacetate 3-hydroxylase, from Sulfobacillus acidophilus TPY
Source: BMC Microbiol. 2019 Feb 13;19:40. doi: 10.1186/s12866-019-1415-9 (PMC6375179; doi:10.1186/s12866-019-1415-9)

**
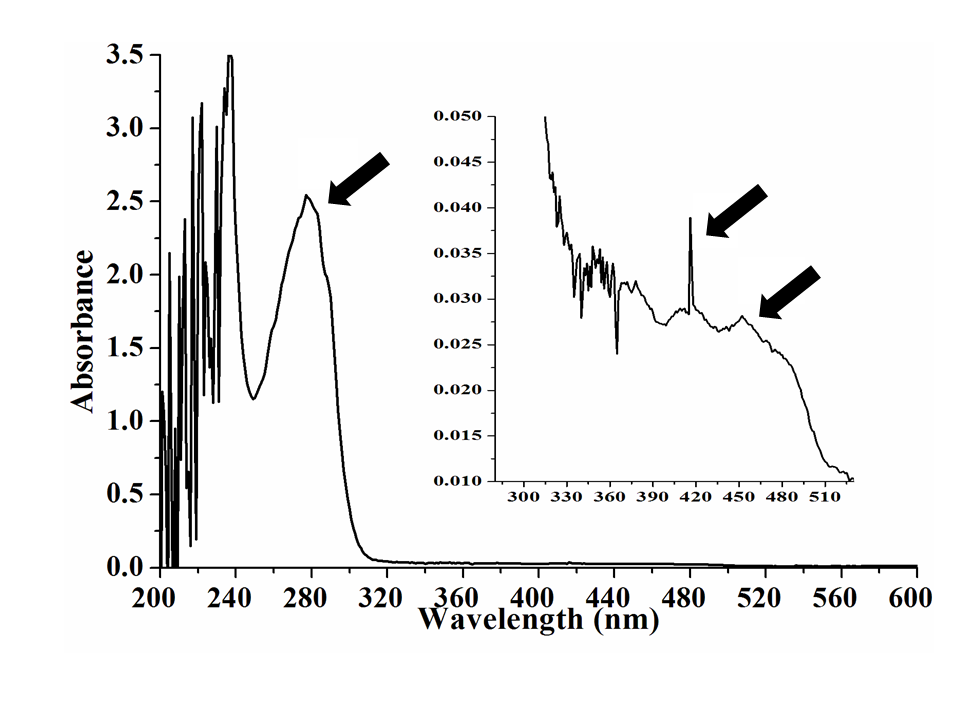
**

**Fig. S2.** UV-visible absorption spectrum of HpaC.

Supplement: Supplementary file 2 — Figure S2. UV-visible absorption spectrum of HpaC. (DOCX 188 kb) [file 12866_2019_1415_MOESM2_ESM.docx]

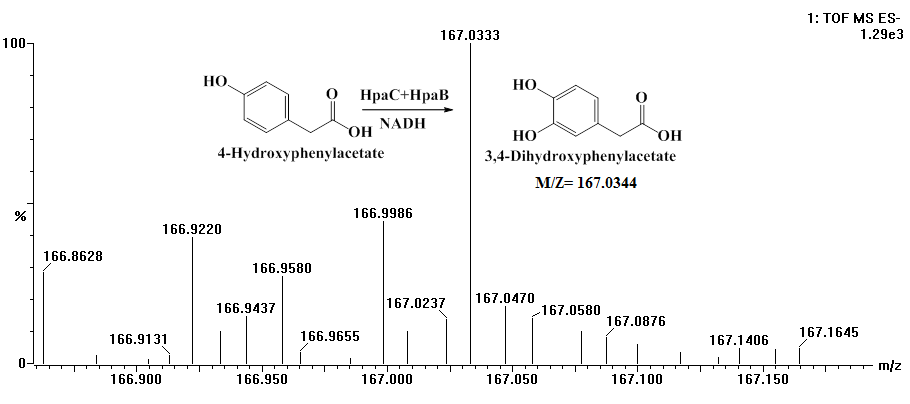


**Fig. S4.** Mass spectrum of 3,4-DHPA transformed from 4-HPA by 4-HPA 3-hydroxylase.

Supplement: Supplementary file 5 — Figure S4. Mass spectrum of 3,4-DHPA transformed from 4-HPA by 4-HPA 3-hydroxylase. (DOCX 77 kb) [file 12866_2019_1415_MOESM5_ESM.docx]
