# Supplementary material for: Characterization of enzymatic properties of two novel enzymes, 3,4-dihydroxyphenylacetate dioxygenase and 4-hydroxyphenylacetate 3-hydroxylase, from Sulfobacillus acidophilus TPY
Source: BMC Microbiol. 2019 Feb 13;19:40. doi: 10.1186/s12866-019-1415-9 (PMC6375179; doi:10.1186/s12866-019-1415-9)
Supplement: Supplementary file 3 — Figure S3. SDS-PAGE analysis of purified proteins. (DOCX 891 kb) [file 12866_2019_1415_MOESM3_ESM.docx]

**
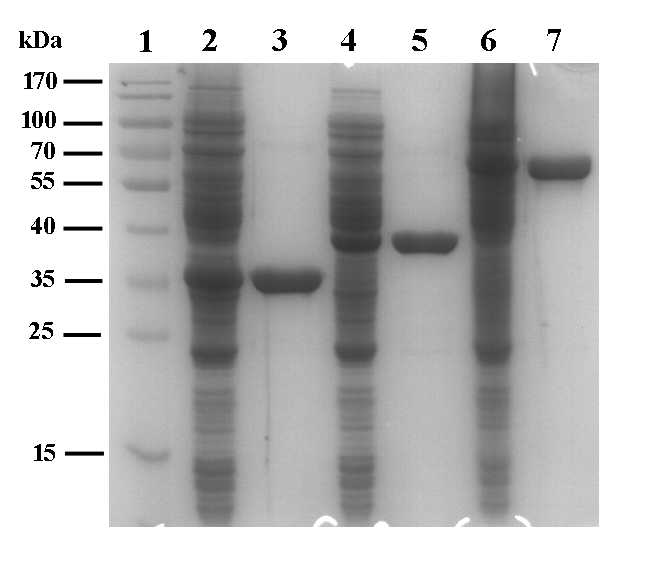
**

**Fig. S3.** SDS-PAGE analysis of purified proteins.

lane 1, molecular weight marker (Takara, Dalian, China); lane 2, supernatant of *E. coli* BL21(DE3) harboring pET-32a(+)-hpaC; lane 3, purified HpaC; lane 4, supernatant of *E. coli* BL21(DE3) harboring pET-28a(+)-mhpB2; lane 5, purified MhpB2; lane 6, supernatant of *E. coli* BL21(DE3) harboring pET-32a(+)-hpaB; lane 7, purified HpaB.
