# Supplementary material for: Characterization of enzymatic properties of two novel enzymes, 3,4-dihydroxyphenylacetate dioxygenase and 4-hydroxyphenylacetate 3-hydroxylase, from Sulfobacillus acidophilus TPY
Source: BMC Microbiol. 2019 Feb 13;19:40. doi: 10.1186/s12866-019-1415-9 (PMC6375179; doi:10.1186/s12866-019-1415-9)
Supplement: Supplementary file 4 — Table S1. The effect of metal ions on DHPAO activity. (DOCX 17 kb) [file 12866_2019_1415_MOESM4_ESM.docx]

Table S1. The effect of metal ions on DHPAO activity.

| Ions | Concentration (M) | Relative activity (%) |
| --- | --- | --- |
| Control | - | 100 |
| AgNO_3_ | 10^-5^ | 93.17± 2.02 |
| CoCl_2_·2H_2_O | 10^-4^ | 90.58± 3.51 |
| BaCl_2_·2H_2_O | 10^-4^ | 103.57± 4.36 |
| Pb(NO_3_)_2_ | 10^-4^ | 131.16± 6.11 |
| MnCl_2_·4H_2_O | 10^-4^ | 19.05± 0.45 |
| MgCl_2_.6H_2_O | 10^-4^ | 112.01± 8.07 |
| CuCl_2_.·2H_2_O | 10^-4^ | 6.37± 0.56 |
| NiSO_4_·6H_2_O | 10^-4^ | 114.18± 0.77 |
| FeCl_3_·6H_2_O | 10^-4^ | 114.03± 0.77 |
| CaCl_2_ | 10^-4^ | 127.48± 7.20 |
| ZnCl_2_ | 10^-4^ M | 112.22± 0.09 |
| Al_2_(SO_4_)_3_·16H_2_O | 10^-4^ | 67.13± 0.18 |
| FeSO_4_·7H_2_O | 10^-4^ | 92.34± 0.88 |
